# Supplementary material for: Association of Lifelong Intake of Barley Diet with Healthy Aging: Changes in Physical and Cognitive Functions and Intestinal Microbiome in Senescence-Accelerated Mouse-Prone 8 (SAMP8)
Source: Nutrients. 2019 Aug 1;11(8):1770. doi: 10.3390/nu11081770 (PMC6723110; doi:10.3390/nu11081770)
Supplement: Supplementary file 1 [file nutrients-11-01770-s001.pdf]

	Rice diet (g/kg diet)	Barley diet (g/kg diet)	Rice diet:Barley diet=1:4 (g/kg diet)
Protein	174	174	174
Fat	120	120	120
Sugar	543	544	544
Dietary fiber	50	79	73

Table S1 Nutrient composition of experimental diets
